# Characterization of a core region in the A2UCOE that confers effective anti-silencing activity

**DOI:** 10.1038/s41598-017-10222-3

**Published:** 2017-08-31

**Authors:** Fang Zhang, Giorgia Santilli, Adrian J. Thrasher

**Affiliations:** Molecular and Cellular Immunology, UCL GOS Institute of Child Health, London, WC1N 1EH UK

## Abstract

We have previously shown that reliability of the A2UCOE in driving transgene expression can be attributed to its resistance to DNA methylation, and its ability to confer this property to linked regulatory sequences. In order to gain a better understanding of how resistance to DNA methylation from the A2UCOE is conferred, and whether the anti-silencing effect from the A2UCOE is confined within a core region, we evaluated the anti-silencing effect of different sub-domains. We found that maximal epigenetic regulatory activity was contained within a 455 bp element derived from the CBX3 region when tested in the context of a lentiviral vector in murine embryonic stem (ES) cells and human inducible pluripotent stem (iPS) cells. This region possessed an active chromatin signature, and operated effectively in *cis* to protect linked heterologous regulatory elements from methylation, thereby conferring stable transgene expression. Defined UCOE elements may be particularly useful for use in vectors where gene expression is desired in methylation-prone chromatin environments such as those encountered in pluripotent stem cells.

## Introduction

Levels of expression from integrated transgenes are strongly influenced by the surrounding chromatin environment. Epigenetic marks including DNA methylation and histone deacetylation promote chromatin condensation, which is generally associated with lower levels of gene transcription^1–5^. The A2UCOE is a methylation-free CpG island extending over divergently transcribed promoters derived from a human housekeeping gene locus associated with the chromobox protein homolog3 (CBX3) and the heterogeneous nuclear ribonucleoproteins A2B1(HNRPA2B1)^6, 7^. It has been demonstrated that the A2UCOE region acts as a dominant chromatin remodelling domain, which is sufficient to prevent transcriptional silencing or variegated expression even when the transgene is integrated within centromeric heterochromatin^6, 7^. To overcome transgene silencing in the context of vector-mediated gene transfer, we have previously incorporated a 2.2 kb A2UCOE sequence into lentiviral vectors, and demonstrated stable transgene expression in hematopoietic cells *in vitro* and *in vivo*, including successful rescue of the X-linked severe combined immunodeficiency (SCID-X1) phenotype in a mouse model^8^. Since then, several other studies have also demonstrated the effectiveness of the A2UCOE for regulation of therapeutic genes in alternative models^9–11^.

By applying DNA methylation analysis *in vitro* and *in vivo* following lentiviral gene transfer, we have shown that the stability of transgene expression from the A2UCOE is due to its resistance to DNA methylation-mediated gene silencing^12^. We have also demonstrated that linking a 1.2 kb sub-fragment of the A2UCOE upstream of a gammaretroviral Long Terminal Repeat (LTR) can negate DNA methylation from this element and lead to stable transgene expression in mouse embryonic carcinoma P19 cells. This finding suggested that the sequences responsible for the anti-silencing function of the A2UCOE could be further refined, and studies have subsequently defined different A2UCOE regions in combination with heterologous promoters in order to achieve stable transgene expression^13–16^.

Using promoter truncation analysis in mouse embryonic carcinoma cells, a recent study has revealed that methylation autonomy in promoter sequences often depends on small critical methylation-determining regions (MDR). MDR regions in most cases harbour the *cis-regulatory* information sufficient for directing hypomethylation^17^. In order to gain a better understanding of how a resistance to DNA methylation from the A2UCOE is conferred, and whether the anti-silencing effect from the A2UCOE is defined within a small critical region, we have evaluated the anti-silencing effect of different sub-domains. We found that a 455 bp fragment derived from the CBX3 region gave rise to the most effective anti-silencing activity in mouse ES cells and human inducible pluripotent stem (iPS) cells. Moreover, we have shown that unlike the adjacent regions, the 455 bp UCOE region retains active chromatin marks and can confer this epigenetic signature to linked heterologous elements.

## Results

### The 455UCOE region is a core region within the 2.2 kb A2UCOE preventing methylation mediated transgene silencing from heterologous regulatory elements

**Figure 1:**
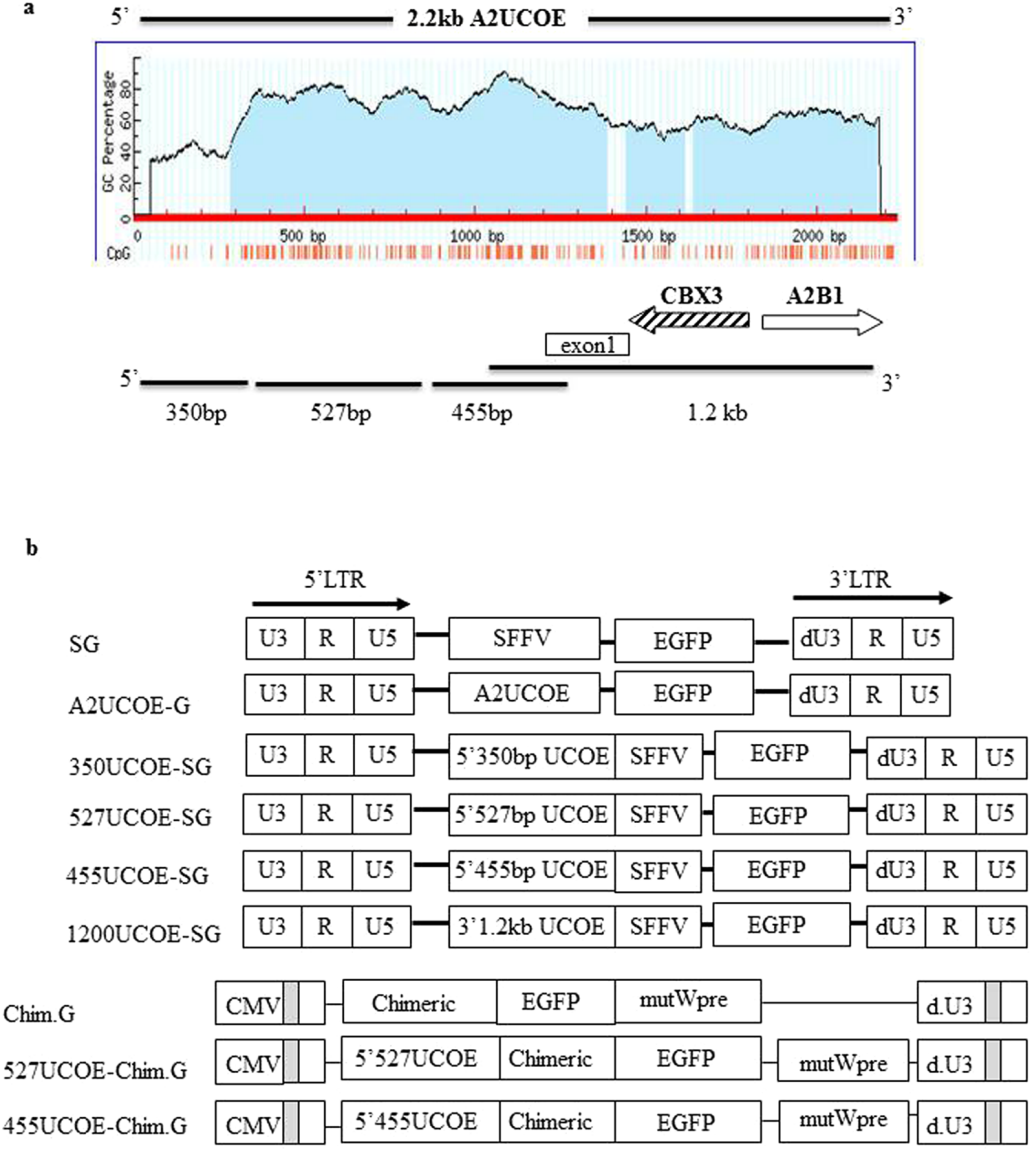
Illustration of lentiviral vectors used in the study. (**a**) The diagram of predicted CpG island in the 2.2 kb A2UCOE using MethPrimer program (www.urogene.org) and corresponding UCOE fragments used in the study. The location of the exon1 of CBX3 is indicated. (**b**) Illustration of the lentiviral vector constructs. LTR: long-terminal repeat; SFFV: Spleen focus-forming virus LTR; CMV: Cytomegalovirus; mutWpre: mutated Woodchuck hepatitis posttranscriptional regulatory element.

**Figure 2:**
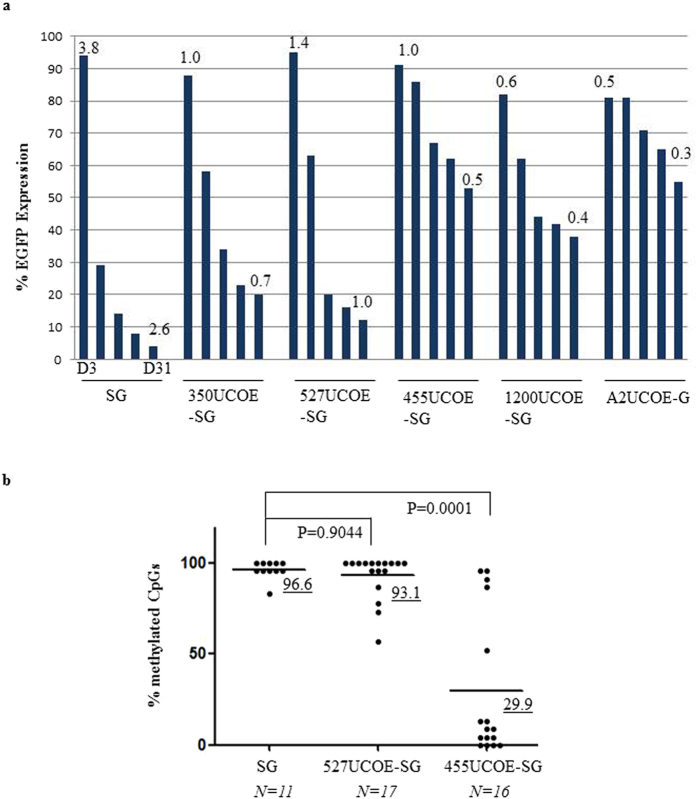
The 455UCOE is a core region in the 2.2 kb A2UCOE element preventing the transgene silencing from the SFFV LTR in murine embryonic carcinoma P19 cells. (**a**) EGFP expression in P19 cells following the viruses transduction. P19 cells were transduced with vectors shown in Fig. 1b, at multiplicity of infection (MOI) of 10. EGFP expression was assessed by flow cytometry at different time points up to 31 days. The numbers above each bar represent vector copy number per cell as determined by Q-PCR. (**b**) DNA methylation analysis of the SFFV LTR region. Genomic DNA from cells transduced with the SG, the 527UCOE-SG and the 455UCOE-SG vectors was isolated at day 17 post transduction and subjected to methylation analysis by bisulfite conversion and sequencing. The percentages of methylated CpGs to the total of CpGs on the SFFV LTR from randomly selected PCR colonies for each vector are shown. The underlined numbers are the mean of % methylated CpGs from each vector. *P* values shown are determined by Mann-Whitney U-test (two tailed).

We isolated three consecutive fragments (350 bp, 527 bp and 455 bp) derived from the CBX3 region of the A2UCOE (Fig. 1a). Among them, the 527 bp and the 455 bp region share a similar high density of CpGs (from 60% to 80%). In contrast, the 350 bp region is relatively devoid of CpGs. These sub-fragments were cloned into a lentiviral vector up-stream of the Spleen Focus Forming Virus (SFFV) LTR in both 5′ (5′UCOE) and 3′ (3′UCOE) orientations, and compared to the original SFFV-EGFP and full length A2UCOE-EGFP vectors for stability of gene expression over time (Fig. 1b). These vectors were tested in mouse embryonic carcinoma P19 cells, which provide a strong methylation-prone environment. EGFP expression was assessed by flow cytometry for up to 31 days of culture after transduction. Initial experiments showed that EGFP expression from all the 3′UCOE constructs was less stable compared to their 5′ counterparts except for the 3′1.2 kb A2UCOE construct, and were therefore not further evaluated (data not shown). A time course of EGFP expression from the 5′UCOE constructs and the 3′1200UCOE construct, compared to the SFFV-EGFP and the A2UCOE-EGFP vectors was evaluated in P19 cells transduced at a MOI of 10 (Fig. 2a, Supplementary Fig. S1). EGFP expression from the SFFV LTR alone declined rapidly (from 94% to 4% at day 31). Among the vectors incorporating different UCOE sub-fragments, the 455UCOE-SG gave rise to the most stable EGFP expression (from 91% to 53%), which is similar to that observed with the full length A2UCOE-EGFP vector (from 81% to 55%). The 1200UCOE-SG gave rise to less stable EGFP expression (from 82% to 38%) than that of the 455UCOE-SG. Interestingly, the 527UCOE fragment, which shares the same high density of CpGs as the 455UCOE (Fig. 1a) did not confer stability (from 95% to 12% at day 31). The CpG-poor 350UCOE-SG vector exhibited a similar expression profile to that of the 527UCOE-SG.

**Figure 3:**
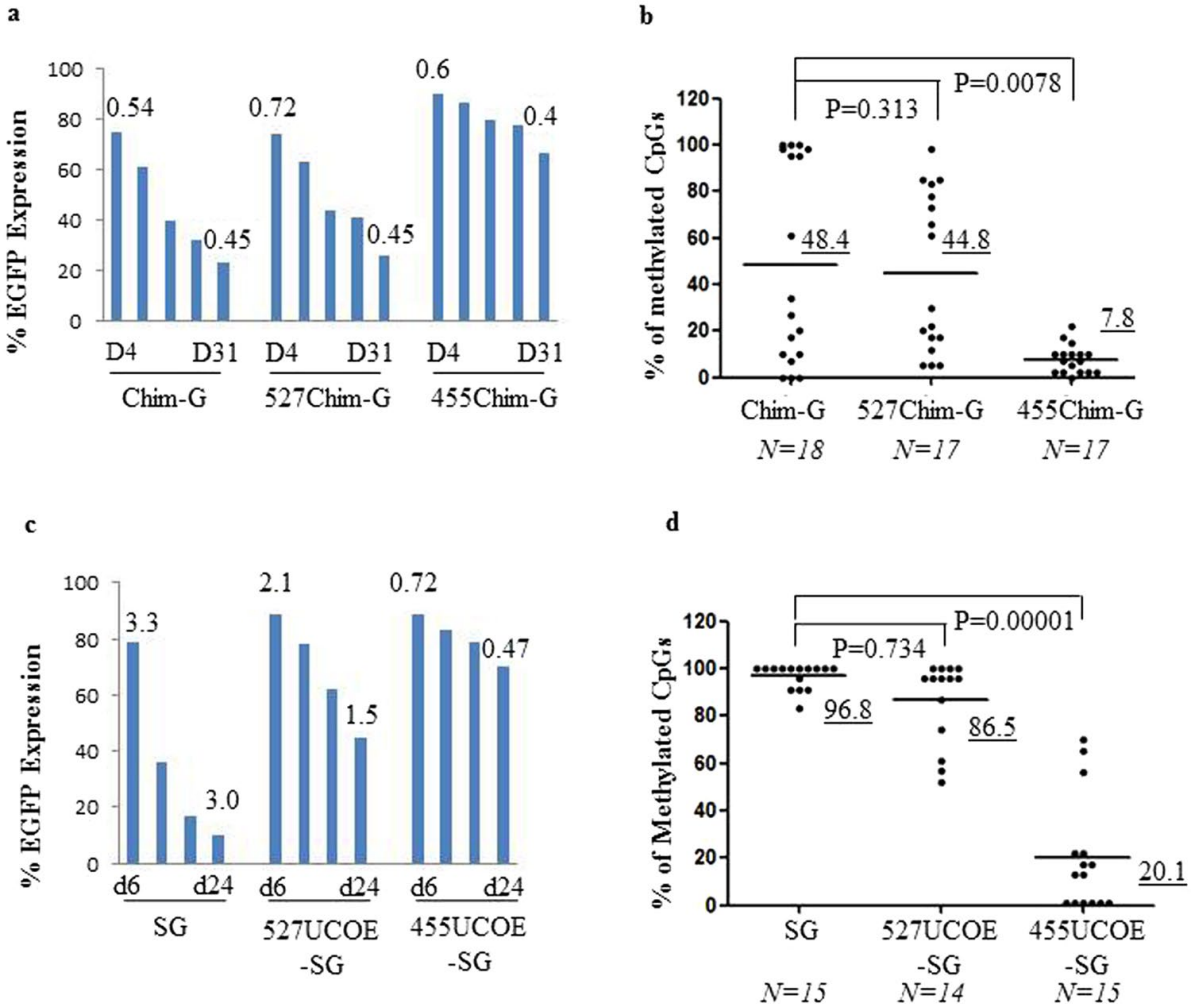
The 455UCOE confers stability to the heterologous promoters in murine embryonic carcinoma P19 cells and in human iPS cells. (**a**) EGFP expression in P19 cells following transduction with the Chim.G, the 527UCOE-Chim.G and the 455UCOE-Chim.G vectors at MOI of 7. (**b**) DNA methylation analysis on the chimeric promoter at day 23 post-transduction. The data is shown as the percentage of methylated CpGs in the total of 41 CpGs on the chimeric region determined as described on the Fig. 2b. (**c**) EGFP expression in human iPS cells following transduction by the SG, the 527UCOE-SG, the 455UCOE-SG vectors. (**d**) DNA methylation analysis on the SFFV LTR at day 24 post-transduction in human iPS cells. The vector copy number per cell is shown above the bars. The underlined numbers are the mean of % methylated CpGs for each vector. *P* values shown determined by Mann-Whitney U-test (two tailed).

We then assessed DNA methylation of the linked SFFV LTR in cells transduced with the SG, the 455UCOE-SG and the 527UCOE-SG vectors at day19 (Fig. 2b, Supplementary Fig. S2). High levels of DNA methylation were present in the SG vector among randomly selected PCR clones of integrated vector copies. A similar methylation pattern was observed for the 527UCOE-SG vector. In contrast, a significantly lower fraction of methylated CpGs was found in the 455UCOE-SG vector. To understand whether the 455UCOE can also prevent transgene silencing from other linked regulatory elements, we placed the 455UCOE and the 527UCOE upstream of a chimeric promoter derived from human *Cathepsin G* and *c-Fes* ^18^ genes (Fig. 1b). EGFP expression from the chimeric promoter declined from 75% to 23% at day31 post-transduction (Fig. 3a). Incorporation of the 527UCOE failed to confer stability of gene expression to the chimeric promoter, showing a similar pattern of EGFP expression (from 75% to 26%) to that of the native chim.G vector. In contrast, the 455UCOE-chim.G vector gave rise to the most stable EGFP expression. DNA methylation at the chimeric promoter sequence among those vectors is shown (Fig. 3b). The mean fraction of methylated CpGs for the chim.G vector from random selected PCR colonies was 48.4%. A similar fraction of methylated CpGs was present at the 527UCOE-chim.G vector. In contrast, a significant lower fraction of methylated CpGs (7.8%) was found at the 455UCOE-chim.G vector.

Lentiviral vectors remain subject to a certain degree of DNA methylation-mediated silencing, or variegated position effect in inducible pluripotent stem cells (iPSc). To see if our observations in mouse P19 cells were recapitulated in iPSc, we tested the different constructs in iPSc derived from human fibroblasts. The same result was obtained showing that the 455UCOE, not the 527UOCE, consistently enables stable EGFP expression from the SFFV LTR due to abrogation of DNA methylation (Fig. 3c,d). Taken together, our data demonstrated that the 455UCOE region is a core DNA MDR within the A2UCOE, and acts in *cis* to regulate *de novo* DNA methylation on adjacent heterologous regulatory elements.

### The 455 bp UCOE retains active chromatin domain

**Figure 4:**
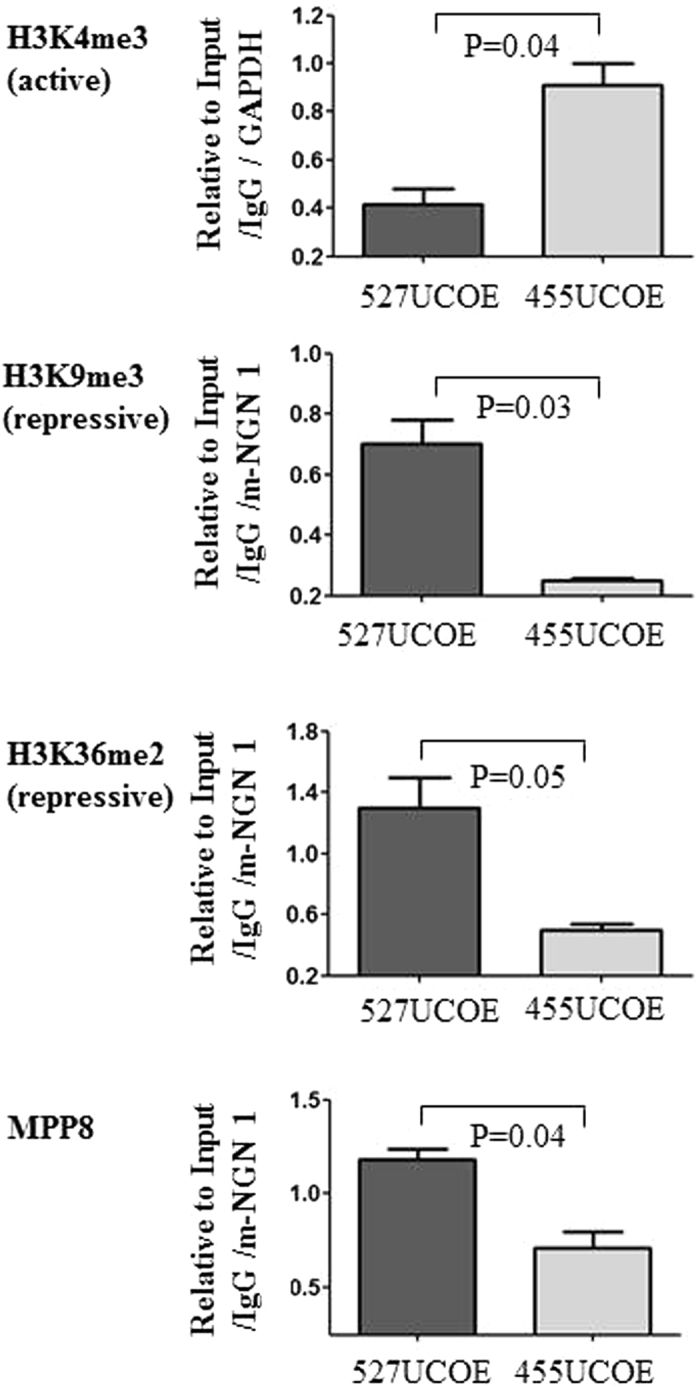
The 455UCOE region remains as an active chromatin domain compared to the 527UCOE region in murine P19 cells. Enrichment of the active and repressive histone marks and the chromodomain protein MPP8 on the 455UCOE and the 527UCOE region was assessed by ChIP assay in transduced P19 cells at day 10 post transduction. The value of enrichment for each antibody is determined relative to the input, IgG and then normalized to the actively transcribed GAPDH or to the repressive NGN1 locus. The normalized values of the enrichment at the 527UCOE, and the 455UCOE are shown. The data represents 2–3 independent ChIP assays. *P* values shown are determined by the *t*-test (two tailed).

Since the 455UCOE region and the 527UCOE region contain a similarly high content of CpGs, but showed a divergent activity in regulation of DNA methylation when linked with other regulatory elements, we tested whether the two regions possessed different epigenetic marks. For this, we performed chromatin immunoprecipitation (ChIP) assays on P19 cells at day 10 and day 17 after transduction, to assess the enrichment of the active histone mark H3K4me3, and the repressive marks H3K9me3, H3K36m2. The enrichment of the chromodomain protein MPP8 (M phase phosphoprotein 8) was also assessed as this protein is known to play a role in deposition of the repressive mark H3K9me3 by recruiting the H3K9 methyltransferase^19, 20^. At d10 post-transduction, the enrichment of the active histone mark H3K4me3 was 2.1 fold higher within the 455UCOE region (0.92 ± 0.12) normalized to actively transcribed GAPDH, compared with the 527 UCOE region (0.43 ± 0.06). In contrast, the enrichment of the repressive histone marker H3K9me3 was 2.8 fold higher at the 527UCOE region (0.7 ± 0.11) compared to the 455UCOE region (0.25 ± 0.01) normalized to the repressive NGN-1 locus (Fig. 4, Supplementary Fig. S3). Interestingly, this high level of H3K9me3 correlated with a high occupancy of MPP8 at the 527UCOE (1.25 ± 0.06) compared to the 455UCOE (0.71 ± 0.12). Similarly, enrichment of the repressive mark H3K36me2 normalized to NGN-1 was 2.6-fold higher at the 527UCOE (1.3 ± 0.08) than at the 455UCOE (0.5 ± 0.06). A similar pattern of enrichment of those marks was obtained at day 17 post- transduction (Supplementary Fig. S4). Overall, our data has revealed a distinct and divergent pattern of histone modification marks within 455UCOE and the 527UCOE regions. A relatively high level of active histone marks and a low level of repressive marks at the 455UCOE region suggest that this element retains an active chromatin configuration, in contrast to the 527UCOE region.

Two CpG binding proteins, Cfp1and KDM2A, have been shown to be important for establishment of non-methylated CpG islands (CGI)^21–24^. Cfp1 recruits H3K4me3 at CGI by forming a complex with the H3k4 methyltransferase Set1. KDM2A, as a demethylase, binds at the CGI, and is responsible for depletion of H3K36me2 specifically. In order to explore the molecular basis underlining the distinctive epigenetic features found on the 455UCOE and the 527UCOE, we performed ChIP assays to assess enrichment of Cfp1 and KDM2A proteins on the two fragments in transduced P19 cells. Surprisingly, we did not find a significant difference in enrichment of the Cfp1 (Supplementary Fig. S5). In contrast, we found a significantly higher level of KDM2a at the 527UCOE region compared to the 455UCOE region (Supplementary Fig. S5). It therefore remains unclear whether these defined CpG-binding proteins play a role in chromatin remodelling at the A2UCOE locus.

### The active chromatin domain from the 455UCOE acts in *cis* regulating epigenetic status on the linked heterologous regulatory sequences

**Figure 5:**
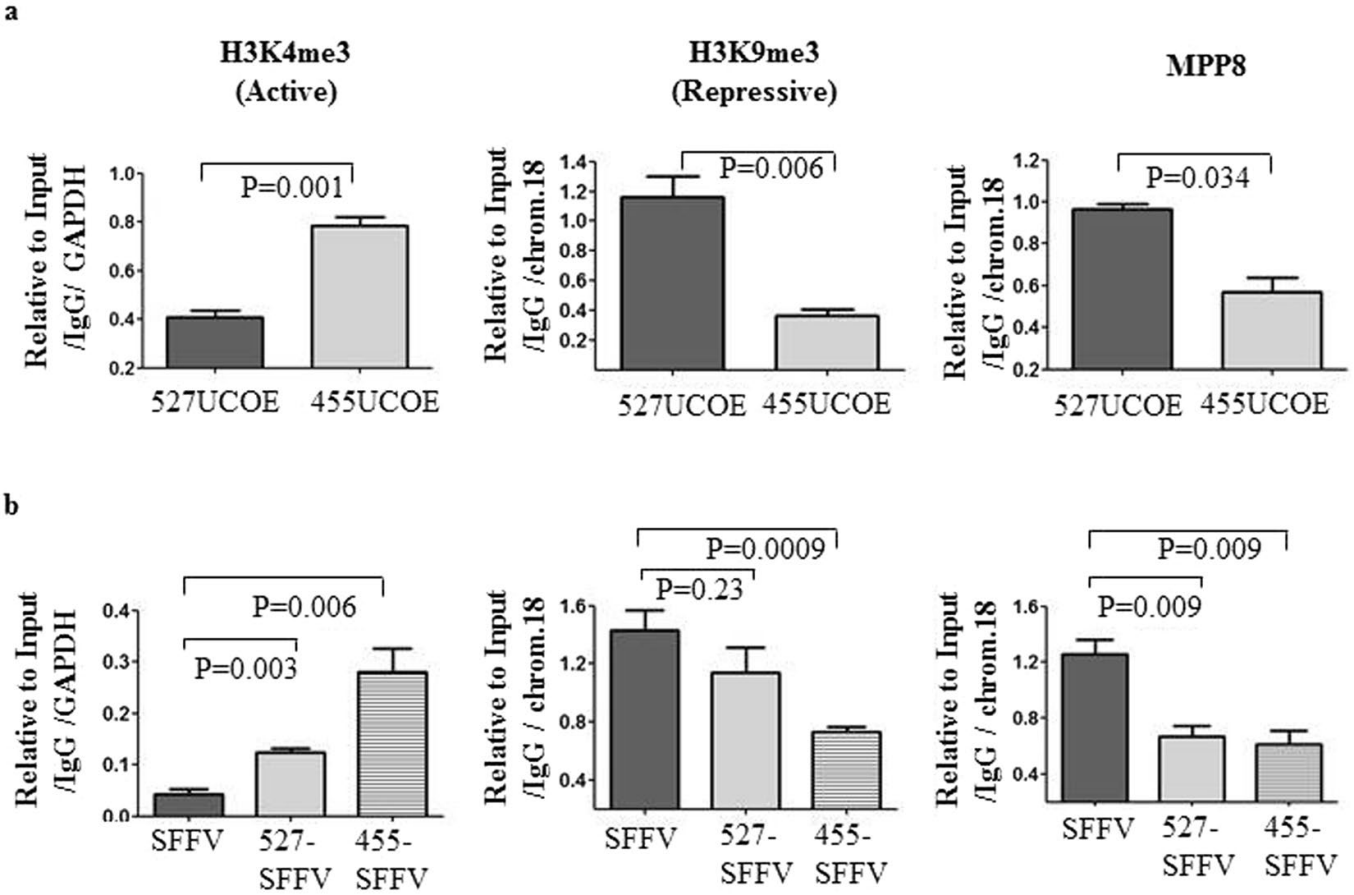
An active chromatin domain from the 455UCOE acts in *cis* regulating epigenetic status on the linked heterologous regulatory sequences. (**a**) Enrichment of histone modification marks and the chromodomain protein MPP8 at the 527UCOE and the 455UCOE regions in transduced hiPS cells was assessed by ChIP assay at day 23 post-tranduction. The value of enrichment for each antibody is determined relative to the input, IgG and normalized to the actively transcribed hGAPDH or to the repressive human chromosome 18 locus. The normalized values of the enrichment at the 527UCOE, and the 455UCOE are shown. (**b**) Enrichment of histone modification marks and the MPP8 at the SFFV LTR in transduced hiPS cells were assessed as above. The normalized values of the enrichment at the SFFV LTR regions are shown. The data represents three independent ChIP assays. *P* values shown are determined by the *t*-test (two tailed).

In order to know if the 455UCOE similarly influences the chromatin signature at adjacent heterologous sequences, we performed ChIP assays to assess the enrichment of active histone marks H3K4me3, repressive histone mark H3K9me3, and the chromodomain protein MPP8 in human iPS cells transduced with the SG, 527UCOE-SG, and 455UCOE-SG after 23 days. The enrichment of histone modification marks at the 527UCOE and the 455UCOE regions were very similar to those obtained previously in P19 cells, showing significant enrichment of active H3K4me3 and a lower level of the repressive H3K9me3 and MPP8 marks on the 455UCOE region (Fig. 5a). Similarly, the active mark H3k4me3 was nearly completely absent at the SFFV LTR when not linked to any UCOE sub-fragment (0.06 ± 0.009) (Fig. 5b). However, this active histone mark increased 5.2 fold high at the SFFV LTR when linked to the 455UCOE (0.31 ± 0.12). A much smaller increase was noted at the SFFV LTR linked to the 527UCOE (0.14 ± 0.07). In contrast to H3k4me3, the repressive mark H3K9me3 was present at a high level on the SFFV LTR alone (1.43 ± 0.23). The level of this repressive mark was significantly reduced at the SFFV LTR linked with the 455UCOE (0.72 ± 0.06), but not at the SFFV linked with the 527UCOE (1.14 ± 0.28). Surprisingly we also found that the level of MPP8 binding was significantly reduced at the SFFV LTR linked to both of the UCOE sub-fragments, despite different levels of H3K9me3, suggesting that other factors may be involved in H3K9me3 marking. Overall our data suggest that an active chromatin signature mediated by the 455UCOE extends to linked heterologous sequences thereby enabling stable transgene expression.

## Discussion

In order to gain a better understanding of how resistance to silencing from the A2UCOE is conferred, and whether there is a core MDR within the A2UCOE, we derived sub-fragments of the region and tested their activity in a methylation prone cell line and iPSCs. We have shown that the 455UCOE sub-fragment derived from the CBX3 of the A2UCOE gave rise to the most effective anti-silencing activity compared to the other regions (Figs 2 and 3). This suggests that a core MDR in the A2UCOE lies within the 455UCOE. To our surprise, the 527UCOE region which shares the same high CpGs density as the 455UCOE exhibited no similar activity. This indicates that the CpG content and density alone does not necessarily contribute to anti-silencing activity. The negative anti-silencing effect of the 527UCOE does not appear to antagonise the function of the 455UCOE as a construct containing both UCOE sequences confers the same anti-silencing effect as that of the 455UCOE alone (data not shown). This further suggests that the 455UCOE region possesses a dominant and independent MDR.

To date, two truncated A2UCOE fragments (the 1.2 kb, and 1.5 kb) have been examined in the context of lentiviral vectors, and when linked to heterologous elements driving transgene expression^7, 8, 14, 15^. The 1.5 kb A2UCOE includes exon1 of A2B1, which is absent from the 1.2 kb A2UCOE (as depicted in Fig. 1a). The anti-silencing effect of both of these A2UCOE sub-fragments has been demonstrated in independent studies. An alternative sub-fragment derived mainly from the CBX3 of the A2UCOE has also been described recently^16^. This fragment was able to prevent silencing of juxtaposed heterologous promoters *in vitro* and *in vivo* by epigenetic remodelling. However, the epigenetic status of this fragment was not investigated. Sequence alignment shows that there is a 327 bp overlap between this and the 455UCOE described in our study, making it likely that the overlap sequences retain key potency of the element. In another study, an alternative 0.7 kb A2UCOE fragment linked to the SFFV LTR was also shown to confer protection against methylation and gene silencing *in vitro* ^13^.

In order to understand mechanisms of the anti-silencing effect, we have characterised the epigenetic signatures on the 455UCOE and 527UCOE sub-fragments. We have found that the 455UCOE possesses a higher level of the active histone mark H3K4me3, and a lower level of repressive histone marks H3K9me3 and H3K36me2, when compared to the 527UCOE (Figs 4 and 5a). We have also demonstrated that the active chromatin domain from the 455UCOE can act in *cis* to confer similar epigenetic changes to linked regulatory elements (Fig. 5b). Therefore, it is likely that the distinctive chromatin signature of these two UCOE regions reflects their different anti-silencing effects. The epigenetic characteristics of the native A2UCOE has been previously characterised in a region ranging from the CBX3 + 6.6 kb to the A2B1 + 7.0 kb in primary human peripheral blood mononuclear cells^25^. Interestedly, the sequences from one CBX3 amplicon identified in that study overlap with part of 455UCOE region. This further confirms that sequences within the 455UCOE can maintain epigenetic autonomy even when placed into vector transgenes and integrated at varying genomic loci in different cell types.

To further explore the distinctive epigenetic features found on the 455UCOE and the 527UCOE, we assessed the contribution of two newly characterised CpG binding proteins, Cfp1^21–23^ and the KDM2A^24^ (Supplementary Fig. S5). However, we did not find evidence for a direct correlation of the enrichment of those CpG binding proteins to the characterised epigenetic marks. Using forward genetic screens, a recent study has revealed a novel human silencing hub complex (HUSH), consisting of three proteins MPP8, TASOR and Periphilin^26^. In this study it was shown that the HUSH complex mediated epigenetic repression through the recruitment of SETDB1 and the deposition of H3K9me3. Since we have observed a relative high enrichment of H3K9me3 and MPP8 on the 527UCOE compared to the 455UCOE (Figs 4 and 5a), we hypothesised that the HUSH complex plays a role in differentiating the epigenetic status of the two UCOE regions. However, we found no significant difference in the enrichment of either TASOR or Periphilin at the two UCOE regions and the SFFV LTR in transduced human iPS cells (data not shown).

In summary, by comparing sub-fragments across the full length A2UCOE sequence, we have demonstrated here that the most effective anti-silencing domain of the A2UCOE lies in a 455bp fragment within the CBX3 promoter region. This region possesses relatively active chromatin marks, and can confer the same epigenetic signature to adjacent heterologous sequences to stabilize downstream transgene expression. This element in a refined format could be potentially useful for prevention of transgene silencing, particularly in cellular contexts where methylation and gene silencing are undesirable.

## Methods

### Generation and production of lentiviral vectors

The 455UCOE-SG vector: a 455 bp Bgl 1/BsrB 1 CBX3 fragment was isolated from previously described Lentiviral A2UCOE-EGFP vector^8^ and ligated into lentiviral SFFV-EGFP vector^8^ at the upstream of the SFFV LTR. The 527UCOE-SG vector: a 527 bp BsrB1/BsrB1 CBX3 fragment was isolated and constructed as the above. The 350 bp UCOE-SG vector: a 350 bp BsrB 1/Kpn1 CBX3 fragment was ligated as the above. The 1200UCOE/SG vector: a BsmB1/BamH1 fragment containing the CBX3 and the A2B1 sequences was constructed as previously described^12^. The 455UCOE-Chim.G, and the 527UCOE-Chim.G vectors were constructed by ligation of the 455 bp, and 527 bp UCOE fragments at the upstream of the Chimeric promoter driving EGFP expression^18^. Lentiviruses were produced as described as before^8^. Briefly, HEK293T cells were co-transfection with three plasmids: the LV pMD.G2 (envelop plasmid) and pCMVΔ8.91 (packaging plasmid), with polyethylenimine (Sigma-Aldrich), and the supernatants were collected at 48 h and 72 h after transfection and concentrated by ultracentrifugation. The titres were determined by the transduction of 293 T cells with the viruses in limiting dilution and EGFP expression was analysed by flow cytometry (Beckman Coulter CyAn ADP) using the software Summit V4.4.

### Maintenance of tissue culture cell lines and the virus transduction

Mouse embryonic teratocarcinoma P19 cells were obtained from the European Collection of Cell Cultures and maintained in alpha MEM medium (Sigma-Aldrich, Poole UK) supplemented with 2 mM Glutamine, 1% nonessential amino acids, 2.5% fetal bovine serum, 7.5% calf serum. HEK293T cells were maintained in DMEM medium (Invitrogen, Paisley UK) containing 10% fetal bovine serum (Sigma-Aldrich). The penicillin and streptomycin were used at10 μg/ml in the above cultures. The hiPSC line generated from human fibroblast cells were cultured in ips-Brew XF human medium (Miltenyi Biotec, UK) on the plates coated with Matrigel (Corning UK). The hiPSCs were passaged twice a week using Passage solution XF (Miltenyi Biotec) containing 2 mM Thiazovivin (Miltenyi Biotec).

Lentivirues were transduced to cultured cells at different multiplicities of infection (MOI). Transduced cells were collected every 3–5 days, and EGFP reporter gene expression was analysed by flow cytometry described as the above.

The vector copy number was determined by Real-time quantitative PCR (Q-PCR) using the Bio-Red CFX system (Bio-Red, Hertfordshire UK). The primers and probe sequences for detecting the lentiviral vector, the endogenous reference mouse Titin and human Albumin sequences are listed in Supplementary Table S1.

### DNA methylation analysis

Genomic DNA was isolated from cells transduced with the lentiviruses using the DNeasy kit (Qiagen, Crawley UK). Sodium bisulfite treatment of genomic DNA was performed to convert unmethylated cytosine to thymine residues using the EpiTect bisulfite kit (Qiagen) according to the manufacturer’s instructions. Then converted genomic DNA was used in PCR. The primers were designed based on converted sequences. A nested PCR was applied to detect the methylation status on the SFFV LTR region covering all 23 CpGs. The primers for the Chimeric region covers the 41 of the total 43 CpG sites. The primer sequences used are listed on the Supplementary Table S1. The single PCR product band was purified using the gel extraction kit (Qiagen), and ligated into the PCR 2.1-TOP vector (Invitrogen), and the colonies were randomly selected for the plasmid DNAs preparation followed by sequencing performed by GENEWIZ (Bishop’s Stortford UK).

### Chromatin Immunoprecipitation

Chromatin immunoprecipitation (ChIP) assays were performed using MAGnify ^TM^ Chromatin Immunoprecipitation system (Invitrogen). Briefly, cells were cross-linked in 1% formaldehyde for 10 min, quenched in 0.125 M glycine for 5 min and lysed in cell lysis buffer. The aliquots of lysate contain 1 × 10^6^/100 µl/tube were quickly freeze in Dry ice and stored in −80 °C. The antibodies (2–10 µg) were coupled to Dynabeads in tubes and rotated end-over-end at 4 °C for 2 h. Chromatin was sheared using a Bioruptor (Diagenode), and diluted at the ratio of 1:10 with the dilution buffer containing 1x Protease inhibitors. The diluted chromatin was then added into the tube containing antibody/Dynabeads, and rotating end-over-end at 4 °C for o/n. The Dynabeads were washed, and the reverse cross-linking was performed to dissociate DNA from protein followed by DNA purification according to the protocol. Semi-quantitate PCR was performed using Power-Up SYBR green (Invitrogen). The primers used are listed in Supplementary Table S1. The antisense primers for the 527UCOE and the 455UCOE amplicons are derived from the adjacent vector sequences (at the same position) to avoid detecting the endogenous homologous UCOE sequences. The PCR was repeated twice times for each ChIP assay. The enrichment for each antibody is determined relative to the input, IgG, and then to the active transcribed GAPDH, or the repressive locus for human chromsoma18 or mouse NGN-1. The data presents at least two independent ChIP experiments. The antibodies were used in ChIP assay as follow: rabbit H3K4me3 (Active Motif), rabbit H3K36me2 (Active Motif), rabbit H3K9me3 (Abcam ab8898), rabbit CGBP (Abcam ab56035), rabbit KDM2A (Abcam ab31739), rabbit MPP8 (Proteintech), rabbit α-periphilin (Abcam ab69569), rabbit α-TASOR (Atlas antibodies HPA006735) and rabbit IgG (Invitrogen, included in MAGnify kit).

### Data availability

The datasets generated or analysed during the current study are available from the corresponding author on reasonable request.

## Electronic supplementary material

supplementary information
