# Supplementary material for: Characterization of a core region in the A2UCOE that confers effective anti-silencing activity
Source: Sci Rep. 2017 Aug 31;7:10213. doi: 10.1038/s41598-017-10222-3 (PMC5578987; doi:10.1038/s41598-017-10222-3)
Supplement: Supplementary file 1 — supplementary information [file 41598_2017_10222_MOESM1_ESM.pdf]

# Supplementary Information

## Characterization of a core region in the A2UCOE that confers effective anti-silencing activity

Fang Zhang, Giorgia Santilli and Adrian J Thrasher*

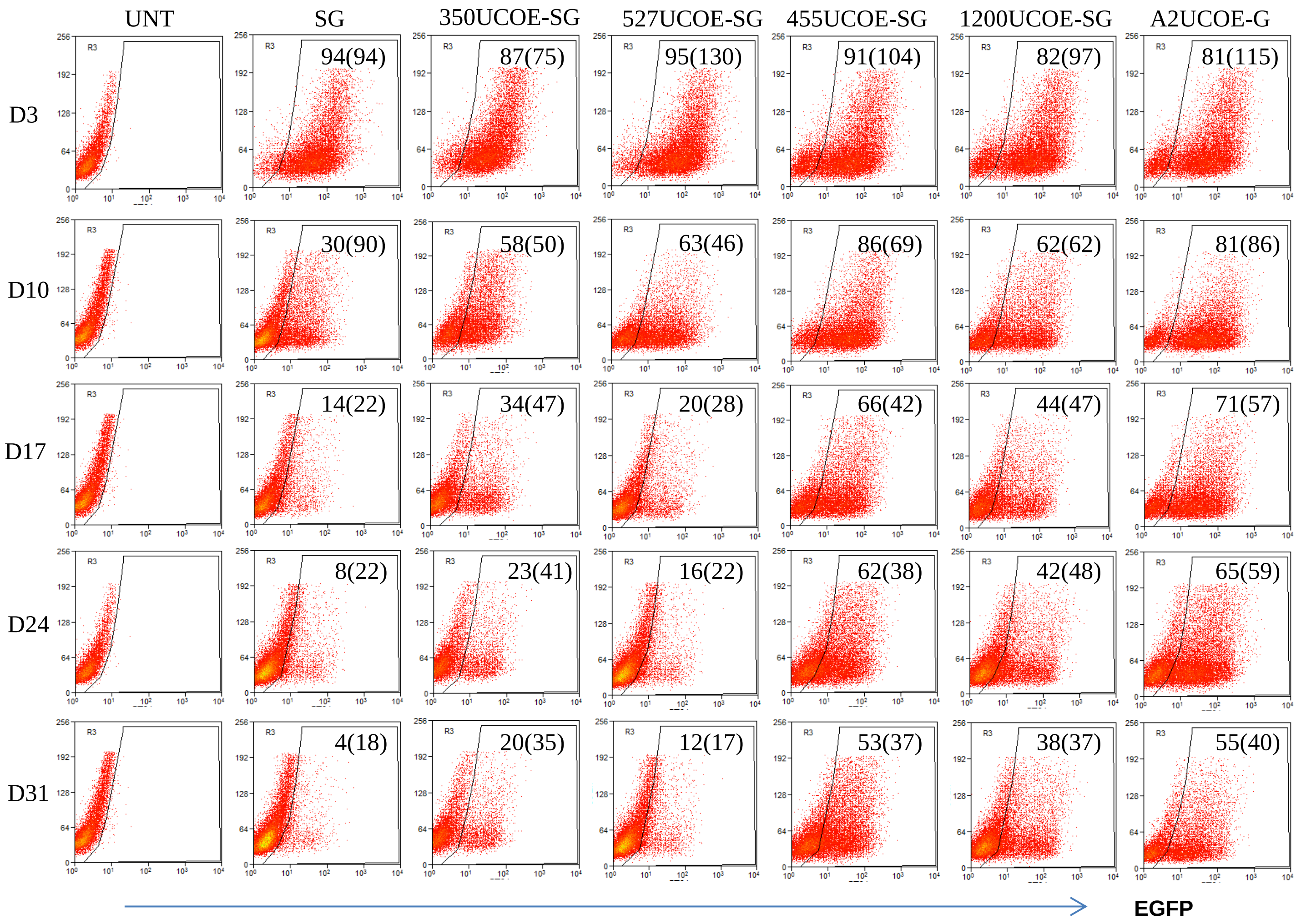


**Supplementary Figure S1: The flow cytometry analysis of EGFP expression in P19 cells following the viral transduction with the indicated vectors at different time points.** The numbers in each plots represent the percentage of EGFP cells and the mean fluorescence intensity (in brackets). UNT: untransduced

SG

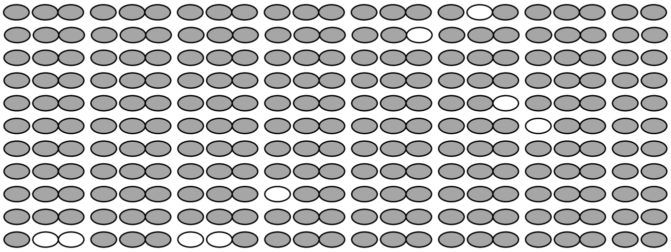

527UCOE-SG

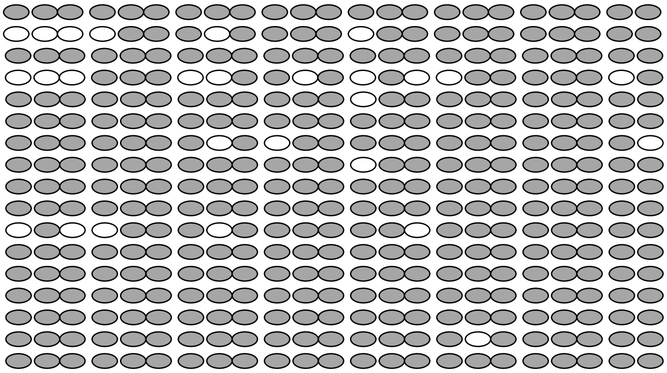

455UCOE-SG

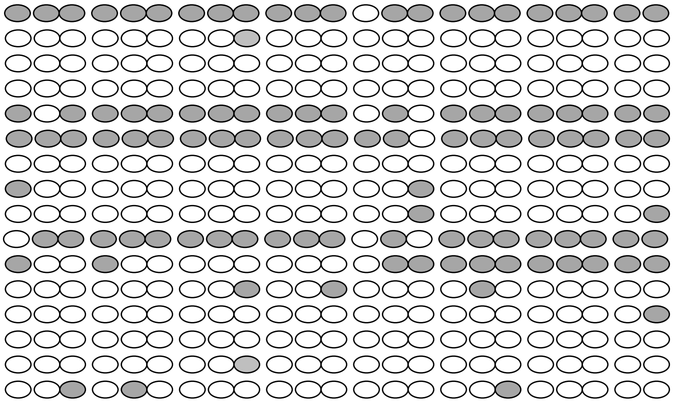

**Supplementary Figure S2**: **The CpG methylation on the SFFV LTR region in P19 cells following the lentiviral transduction.** Genomic DNA from cells transduced with lentiviruses at day 17 was isolated and subjected to methylation analysis by bisulfite conversion and sequencing. Methylation status of randomly selected PCR colonies is shown. White boxes: unmethylated CpG sites. Black boxes: methylated CpG sites. Upper panel: SFFV-EGFP. Middle panel:: 527UCOE-SG. Lower panel: 455UCOE-SG.

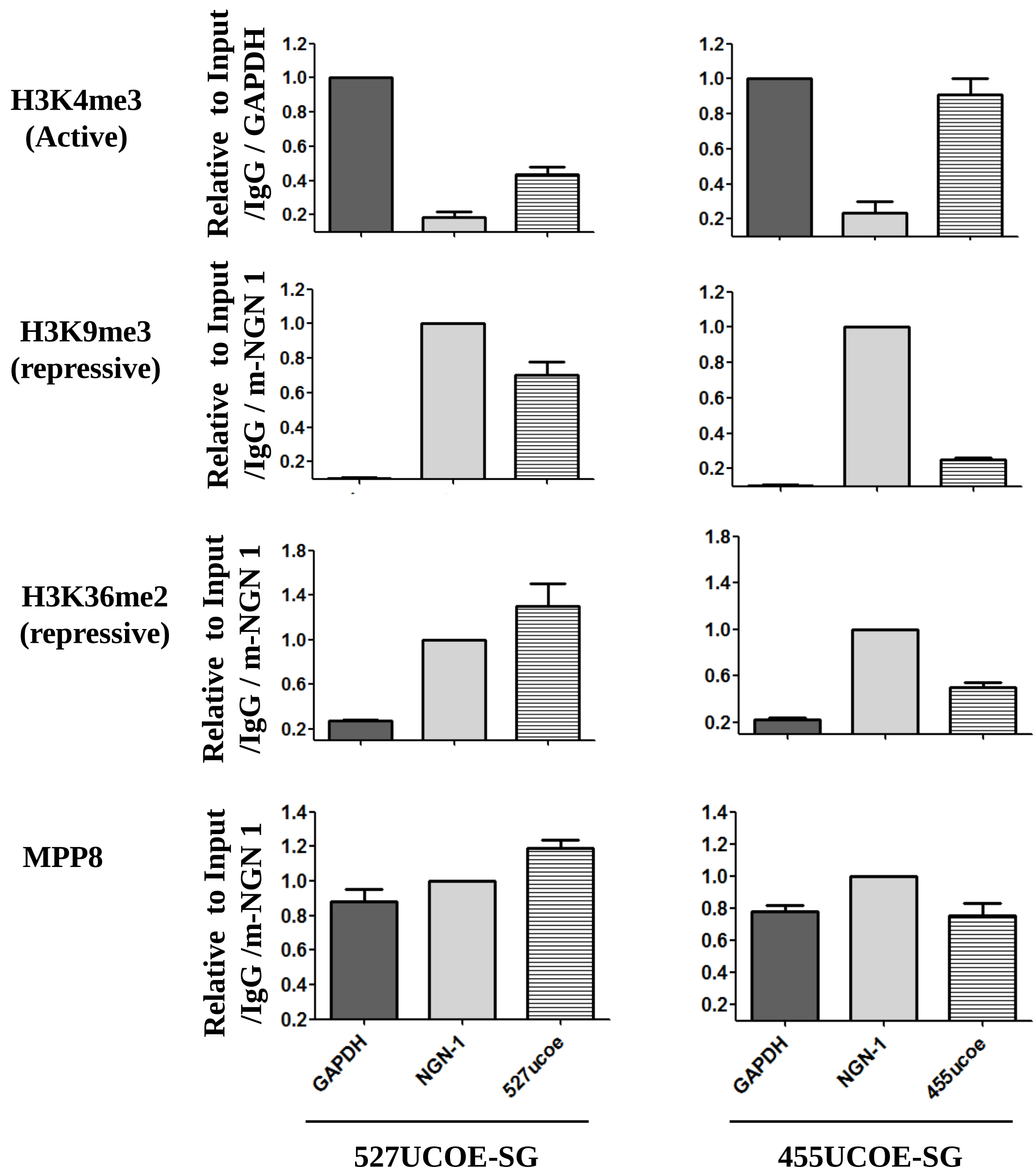


**Supplementary Figure S3**: **The 455UCOE region remains as an active chromatin domain compared to the 527UCOE region in murine P19 cells**. The ChIP assay was performed in P19 cells at day 10 post transduction. The value of enrichment for each antibody is determined relative to the input, IgG and normalized to the actively transcribed GAPDH or to the repressive mouse NGN1 locus. The data represents 2-3 repeated ChIP assays.

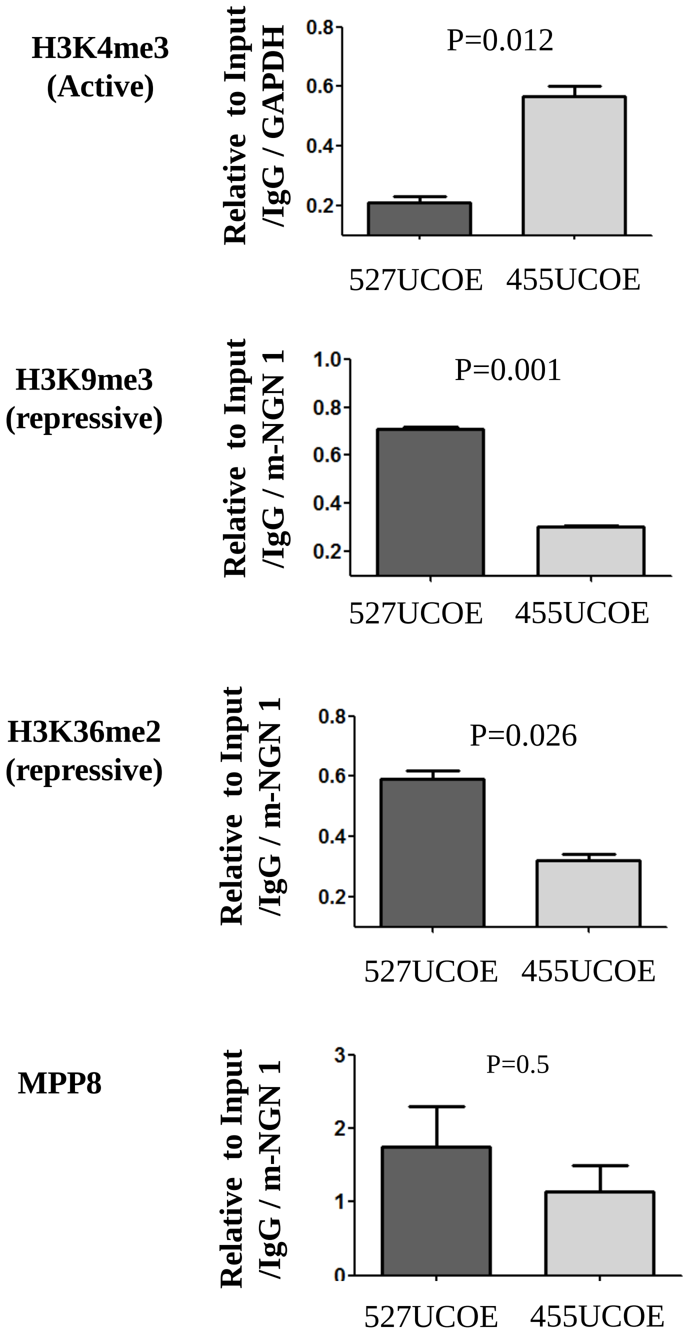


**Supplementary Figure S4**: **The 455UCOE region remains as an active chromatin domain compared to the 527UCOE region in murine P19 cells.** Enrichment of active and repressive histone marks and the MPP8 on the 455UCOE and the 527UCOE region was assessed by ChIP assay in P19 cells at day 17 post transduction. The value of enrichment for each antibodies is determined relative to the input, IgG and then normalized to the actively transcribed GAPDH or to the repressive mouse NGN1 locus. The normalized values of the enrichment at the 527UCOE, and the 455UCOE are shown. The data represents 2-3 independent ChIP assays. *P* values are calculated by Student's *t*-test.

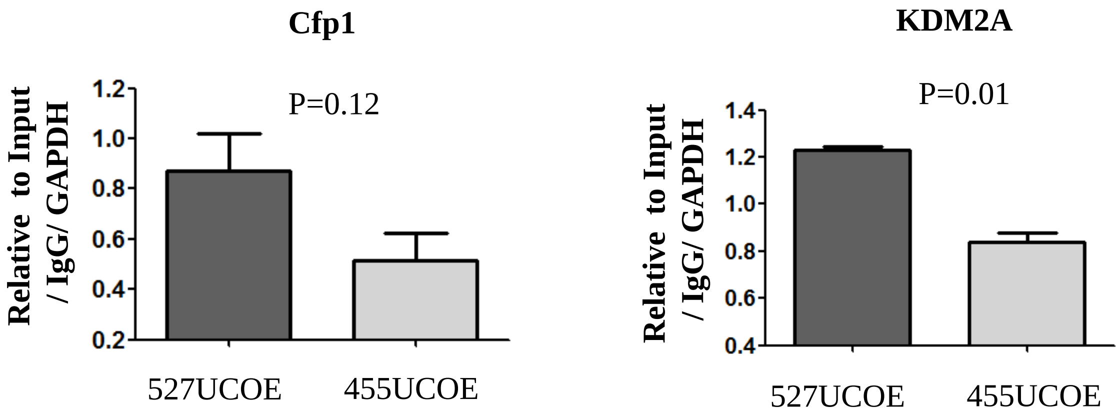


**Supplementary Figure S5: Enrichment of CpG binding proteins Cfp1, KDM2A on the 455UCOE and the 527UCOE regions**. The ChIP assay was performed in P19 cells at day 10 post transduction. The value of enrichment for each antibody is determined relative to the input, IgG and then normalized to the actively transcribed GAPDH. The normalized values of the enrichment at the 527UCOE, and the 455UCOE are shown . The data represents three independent ChIP assay. *P* values are calculated by Student’s *t*-test.

**Supplementary Table S1:** Primers used in DNA methylation, Q-PCR and ChIP analysis.

| Methylation | Forward 5'-3' | Reverse 5'-3' |
|---|---|---|
| Me-SFFV $1_{st}$ PCR | TAGAAAAAGGGGGGAATGAAA | AAACAACTCCTCACCCTTACTCA C |
| Me-SFFV $2_{nd}$ PCR | GGGGGAATGAAAGATTTTATT TG | ACCCTTACTCACCATAATTTC AA CC |
| Chimeric | GGAGGAGGGAGTATAGTAGTA | CAAACCAACTCAAACCCAATAC |
| Q-PCR | | |
| Lenti-PSI | CAGGACTCG GCTTGCTGAAG | TCCCCCGCTTAATACTGACG |
| | Probe: FAM-CGCACGGCA AGA GGCGAGG TAMRA | |
| Mouse Titin | AAAACGAGCAGTGACCTGAGG | TTCAGTCATGCTGCTAGCGC |
| | Probe: FAM TGCACGGAA TCTC GTCTCAGTC TAMRA | |
| Human Albumin | GCTGCTATCTCTTGTGGGCTGT | ACTCATGGGAGCTGCTGGTTC |
| | Probe: VIC-CCT GTC ATG CCC ACA CAA ATC TCT CC TAMRA | |
| ChIP | | |
| 527ucoe | GAAATGCGCTTTGTCTCGAA | CCC CCC TTT TTC TGG AGA CTA* |
| 455ucoe | AGTGACCGGAGTCTCCTC A | CCC CCC TTT TTC TGG AGA CTA* |
| SFFV | AATCAGCCTGCTTCTCGCTTCT | TGA ACA GCT CCT CGC CCT T |
| h-GAPDH | GCTACTAGCGGTTTTACGGGCG | TGC GGC TGA CTG TCG AAC AGG |
| h-Chrom.18 | ACTCCCCTTTCATGCTTCTG | AGGTCCCAGGACATATCCATT |
| m-GAPDH | TCC CCT CCC CCT ATC AGT TC | GAC CCG CCT CAT TTT TGA AA |
| m-NGN-1 | TCCGTTTCCTGCGTTTCAA | TGCTCTGGGCTGGCTGTC |

* See the details in the materials and methods.
